# Longitudinal Assessment of Immune Responses to Repeated Annual Influenza Vaccination in a Human Cohort of Adults and Teenagers

**DOI:** 10.3389/fimmu.2021.642791

**Published:** 2021-03-03

**Authors:** Meng-Hsuan Sung, Ye Shen, Andreas Handel, Justin Bahl, Ted M. Ross

**Affiliations:** ^1^Department of Epidemiology and Biostatistics, College of Public Health, University of Georgia, Athens, GA, United States; ^2^College of Public Health, Health Informatics Institute, University of Georgia, Athens, GA, United States; ^3^Center for the Ecology of Infectious Diseases, University of Georgia, Athens, GA, United States; ^4^Department of Infectious Diseases, College of Veterinary Medicine, University of Georgia, Athens, GA, United States; ^5^Center for Vaccines and Immunology, University of Georgia, Athens, GA, United States

**Keywords:** influenza vaccine, hemagglutination inhibition assay, composite score, repeat vaccination, immune responses

## Abstract

**Background:** The overall performance of a multiple component vaccine assessed by the vaccine-elicited immune responses across various strains in a repeated vaccination setting has not been well-studied, and the comparison between adults and teenagers is yet to be made.

**Methods:** A human cohort study was conducted at the University of Georgia, with 140 subjects (86 adults and 54 teenagers) repeatedly vaccinated in the 2017/2018 and 2018/2019 influenza seasons. Host information was prospectively collected, and serum samples were collected before and after vaccination in each season. The association between host factors and repeated measures of hemagglutination inhibition (HAI) composite scores was assessed by generalized linear models with generalized estimating equations.

**Results:** The mean HAI composite scores for the entire sample (*t* = 4.26, *df* = 139, *p* < 0.001) and the teenager group (*t* = 6.44, *df* = 53, *p* < 0.001) declined in the second season, while the changes in the adults were not statistically significant (*t* = −1.14, *df* = 85, *p* = 0.26). A mixture pattern of changes in both directions was observed in the adults when stratified by prior vaccination. In addition, the regression analysis suggested an interactive effect of age and BMI on the HAI composite scores in the overall population (beta = 0.005; 95% CI, 0.0008–0.01) and the adults (beta = 0.005; 95% CI, 0.0005–0.01).

**Conclusions:** Our study found distinct vaccine-elicited immune responses between adults and teenagers when both were repeatedly vaccinated in consecutive years. An interactive effect of age and BMI on the HAI composite scores were identified in the overall population and the adults.

## Introduction

While there is general agreement that influenza virus vaccines provide some protection against infection, the impact that repeated annual influenza virus vaccination has on the level of protection is still not fully understood (1–5). Hoskins et al. concluded that repeated vaccination did not provide a long-term benefit in reducing the cumulative attack rate when compared with no vaccination and single vaccination (1). However, Keitel et al. reported a contradicting finding showing that repeated annual vaccination resulted in better vaccine efficacy than a single administration (2). Both studies found that subjects' pre-vaccination hemagglutination inhibition (HAI) titers and previous vaccination status significantly influenced their post-vaccination HAI titers (3, 4). Others observed similar levels of vaccine effectiveness among those undergoing vaccination in the current season only and those repeatedly vaccinated in consecutive seasons, implying that repeated vaccination may not have impaired antibody responses (6).

The impact of host factors on the strength of vaccine-elicited immune responses have been widely studied. Several studies showed that aging has an effect on serological responses (7–9). The impact of age on immune response may interact with individual vaccination and infection histories (7, 9). Females had significantly higher antibody titers than males regardless of the influenza strain and dosage of the vaccine (10–12). Interactions between age and sex were also reported, with greater sex differences observed in older adults (13). Obesity is also an important factor correlating with human antibody responses to the influenza vaccine. Several studies found significantly higher initial serologic responses to H1N1 vaccines among obese adults compared to adults of normal weights (14, 15). However, more significant declines in influenza antibody titers and CD8+ T-cell activation post-vaccination were observed among obese participants, indicating the delayed or blunted immune responses against H1N1 in the obese hosts (16).

While sophisticated statistical models have been used in some cohort studies to account for potential within-person dependency (17, 18), only a small fraction of the published studies considered the correlation among the repeatedly measured antibody responses from the same subject. Influenza virus vaccines are often evaluated by the antibody response they elicit, particularly the hemagglutination inhibition (HAI) titers. Current guidelines often evaluate influenza vaccines by each single vaccine component, and HAI titers of 1:40 or greater are considered to be associated with clinical protection (19–21). However, the immune response for a single strain cannot evaluate the overall performance of a multiple component vaccine in consecutive years, given the potential changes of the subtype strains included in the vaccine. To study the overall vaccine-elicited antibody responses from multiple influenza strains, an HAI composite score was proposed to evaluate the serological changes in all 4 vaccine components (22). In this study, we examined the longitudinal changes in the vaccine-elicited immune responses and their associations with host factors in a human cohort with both adults and teenagers.

## Methods

### Study Design

A human cohort vaccine study was initiated in the 2016–2017 influenza season and has been recruiting subjects annually from the Athens, GA, USA metropolitan region in subsequent years. Human sera samples and individual information were collected and tested at the University of Georgia. From 2017–2018 through 2018–2019, eligible subjects, including teenagers (12–18 years) and adults (≥18 years) who had not yet received the seasonal influenza vaccine, were enrolled beginning in September of each year. The details of vaccine formulation and immune response assays have been previously published (22). The influenza strains included in the vaccine formulation for the 2017–2018 season were: A/ Michigan/2015 (H1N1), A/Hong Kong/2014 (H3N2), B/Phuket/2013 (Yamagata-lineage), and B/Brisbane/2008 (Victoria-lineage). In the 2018-2019 season, the vaccine formulation changed to: A/ Michigan/2015 (H1N1), A/ Singapore/2016 (H3N2), B/Phuket/2013 (Yamagata-lineage), and B/ Colorado/2017 (Victoria-lineage).

### Data Collections

Participants who received the standard dose split-virion (IIV) version of licensed FluzoneTM (Sanofi Pasteur, Swiftwater, PA, USA) were selected in this study, with 255 and 242 subjects in the 2017–2018 season and 2018–2019 season, respectively (Supplementary Table 1). Within the 2 consecutive flu seasons, subjects who re-enrolled in the second year would have repeated measurements of immune responses. A total of 140 participants who had assessments of immune responses in both seasons were included in the main longitudinal analysis.

In each influenza season, HAI activity was tested on collected serum samples at Day 0 prior to vaccination and then at Day 21/28 post-vaccination. In addition, individual-level host factors data were prospectively collected. Information on age, sex, race, Body Mass Index (BMI), comorbidity, the month of vaccination, and prior vaccination were collected at first visiting of each year for every participant. The ages of subjects were measured in years. Race included White, Black, American Indian, Asian, and Hispanic. Since over 80% of subjects were Whites, the race variable was eventually dichotomized into White vs. non-White for the subsequent modeling analysis. BMI was calculated by a person's weight in kilograms divided by the square of height in meters. Comorbidity information was self-reported by the participants in each year at enrollment (including diabetes, asthma, hypertension, depression, anxiety, glaucoma, and other uncommon conditions). Day 0 of the 2017–2018 season was considered as our study baseline, and prior vaccination was determined by whether an individual received a flu shot in the 2016–2017 flu season.

### Statistical Analysis

An HAI composite score was created by summing the increasing folds of changes in HAI titers among the 4 influenza strains included in the vaccine formulation in a given year (22). The score was considered as the major outcome assessing overall vaccine-elicited immune responses and was calculated as

$$\begin{array}{l} f\left(HAI\right)={{\left(log_{2}\frac{HAI_{post}}{HAI_{pre}}\right)}_{H1N1}+\left(log_{2}\frac{HAI_{post}}{HAI_{pre}}\right)}_{H3N2}\\ \;+{\left(log_{2}\frac{HAI_{post}}{HAI_{pre}}\right)}_{Bvic}+{\left(log_{2}\frac{HAI_{post}}{HAI_{pre}}\right)}_{Byam}, \end{array}$$

where **HAI**_**pre**_ and **HAI**_**post**_ are HAI titers measured pre- and post- vaccination.

Descriptive statistics on all study participants' demographics were obtained for each flu season. Further, the descriptive statistics for re-enrolled subjects were stratified by flu seasons and age groups (adults vs. teenagers). Box plots were generated for the general population and each age group, stratified by previous vaccination status. Generalized linear models (GLM) with generalized estimating equations (GEE) were then applied to model the associations between the repeated measurements of HAI composite scores and host factors. Covariates included in the model were baseline HAI titers (geometric mean of 4 strains' baseline HAI titers), age, sex, BMI, comorbidity, years (flu season), and prior vaccination history. When included in the regression models, comorbidity was dichotomized by whether an individual possessed at least one comorbidity condition. The final model used the repeatedly measured HAI scores as the outcome and included all covariates and the interaction terms between age and sex, and age and BMI. The compound symmetry correlation structure was adopted in GEE to account for the correlations of repeated measurements from the same subject. In a separate analysis, subjects with HAI composite scores above and below 4 were defined as responders and non-responders, respectively (22). The dichotomized outcome was subsequently modeled by a logistic regression with GEE following the same procedure as the linear model. Subgroup analyses stratified by age were performed by splitting the cohort population into teenagers and adults by a threshold of 18 years old during the 2017–2018 season. The same model settings and scenarios as the overall analysis were then repeated. Additional stratified analyses were conducted in various settings to assess the heterogeneity among different age groups in adults, and to compare repeatedly vaccinated teenagers with those newly enrolled in 2018–2019. All statistical analyses were performed using R software version 4.0.2 (23) with a significance level set at *P* = 0.05.

### Ethics Statement

Study participants were recruited at the UGA Clinical Trials Research Unit (CTRU) in Athens, GA, USA, and enrolled with written, informed consent. The Institutional Review Board of the University of Georgia reviewed and approved the study procedures, informed consent, and data collection documents.

## Results

### Descriptive Analysis

Descriptive statistics on all enrolled participants in our cohort study during the period of 2017–2018 to 2018–2019 are presented in Supplementary Table 1). Overall, white participants contributed over 80% of the total sample size. A noticeable change was that the peak month of receiving vaccination shifted from November of 2017 to September/October of 2018.

**Figure 1:**
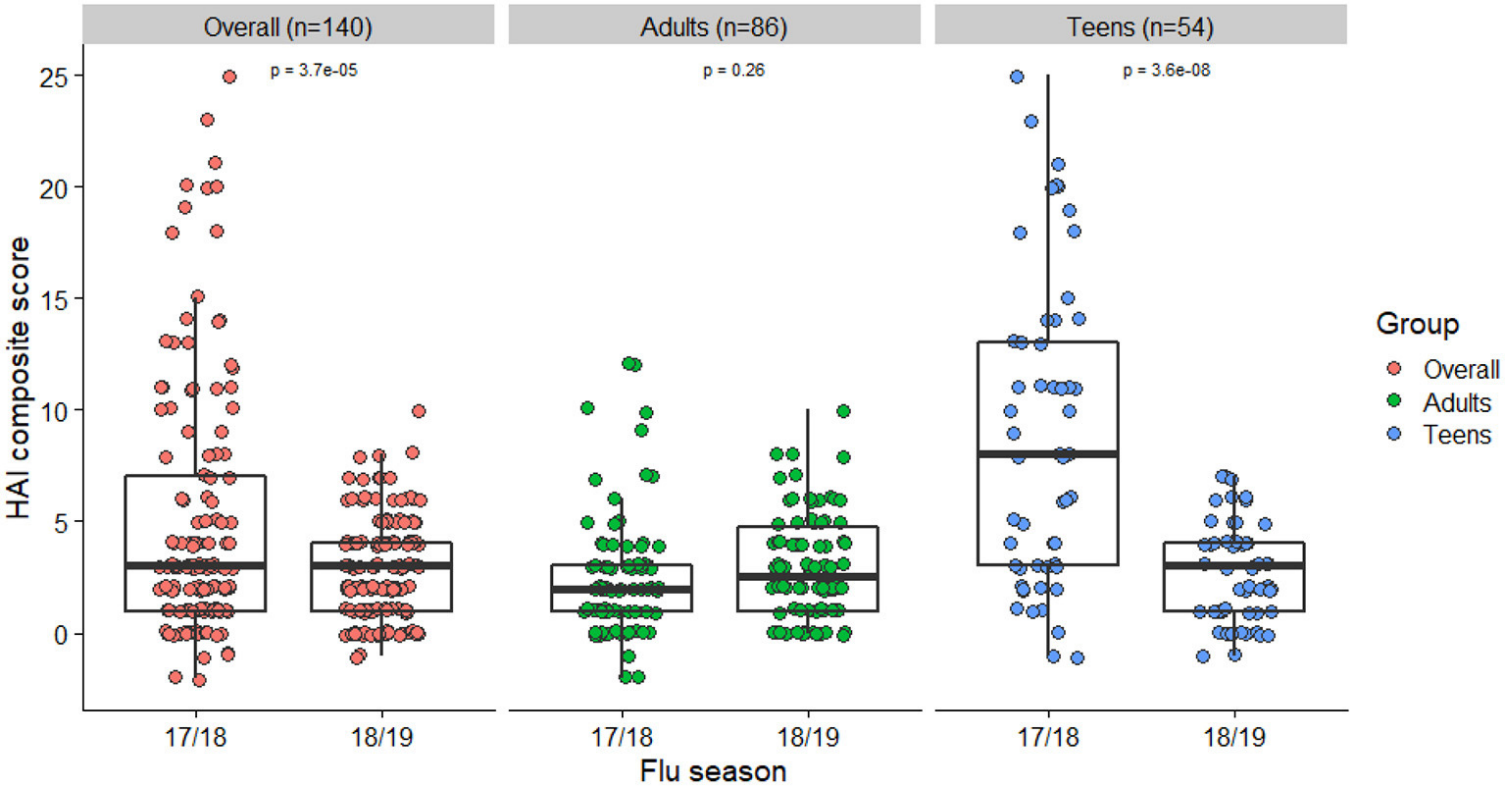
HAI composite scores among different age groups in 17/18 and 18/19 (*N* = 140).

**Figure 2:**
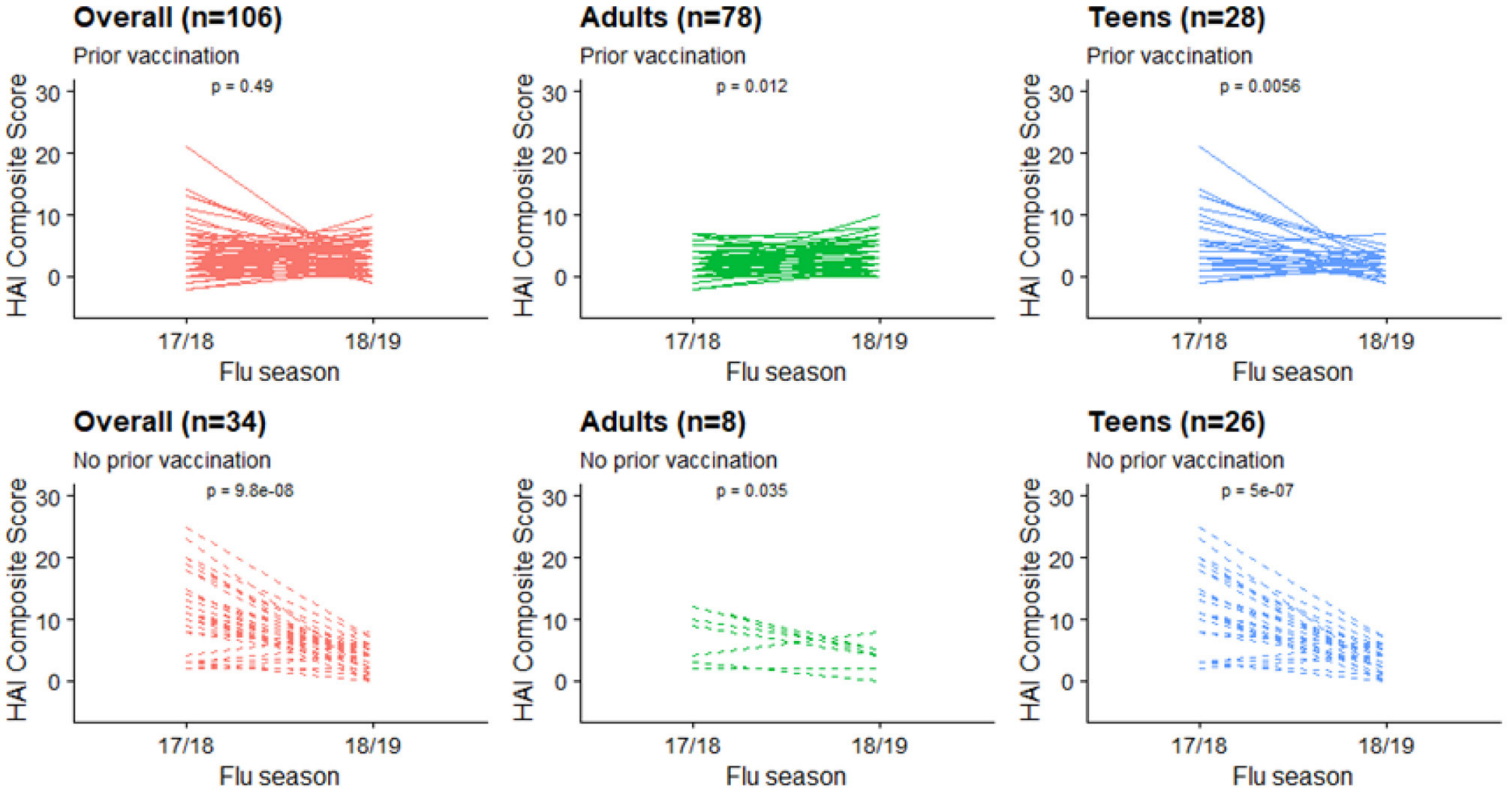
HAI composite scores changes at individual level, stratified by age groups and prior vaccination status.

Characteristics of the 140 subjects used for our main analyses are presented in Table 1. These were participants who were enrolled in the study in both 2017–2018 and 2018–2019 seasons, and thus considered the true cohort repeatedly vaccinated and assessed in both years. Racial composition and patterns of peak vaccination month were similar to the total sample. There were 86 adults and 54 teenagers, respectively. Characteristics were stratified and compared between the 2 groups. Adults had higher BMIs and were more likely to be vaccinated during the previous flu season than the teenagers. A decline in the mean HAI composite score was observed from year 1 (2017–2018) to year 2 (2018–2019). This change was mainly driven by a decline in the teenage group (Figure 1). Their individual-level trajectories of changes were also quite distinct (Figure 2), with a mixture pattern of changes in both directions observed in the adults and a downward trend dominating the teenagers. The decline was also more apparent in those who were not vaccinated in 2016–2017. A subgroup analysis between younger adults (age < 50) and older adults (age ≥ 50) was further considered, and we noticed a difference in average BMI between younger adults and older adults (Supplementary Table 2).

**Table 1:** Descriptive statistics of subjects re-enrolled in both seasons.

	**2017–2018**			**2018–2019**		
	**Overall**	**Adults**	**Teenagers**	**Overall**	**Adults**	**Teenagers**
Sample sizes	140	86	54	140	86	54
Age	29.26 (17.86)	38.73 (16.88)	14.17 (1.24)	30.21 (17.73)	39.67 (16.66)	15.13 (1.29)
Adults (≥18 years)	86 (61.4%)			86 (61.4%)		
Teenagers (<18 years)	54 (38.6%)			54 (38.6%)		
BMI	25.46 (5.62)	27.91 (5.41)	21.58 (3.26)	25.66 (5.56)	27.86 (5.46)	22.16 (3.61)
Sex (Male)	57 (40.7%)	34 (39.5%)	23 (42.6%)	57 (40.7%)	34 (39.5%)	23 (42.6%)
**Race**						
White	114 (81.4%)	69 (80.2%)	45 (83.3%)	114 (81.4%)	69 (80.2%)	45 (83.3%)
African American	6 (4.3%)	5 (5.8%)	1 (1.9%)	6 (4.3%)	5 (5.8%)	1 (1.9%)
Other	20 (14.3%)	12 (14%)	8 (14.8%)	20 (14.3%)	12 (14%)	8 (14.8%)
Comorbidity (Yes)	31 (22.1%)	24 (27.9%)	7 (13%)	36 (25.7%)	22 (25.6%)	14 (25.9%)
**Month of vaccination**						
September	11 (7.9%)	11 (12.8%)	0 (0%)	61 (43.6%)	44 (51.2%)	17 (31.5%)
October	36 (25.7%)	27 (31.4%)	9 (16.7%)	55 (39.3%)	31 (36%)	24 (44.4%)
November	64 (45.7%)	30 (34.9%)	34 (63%)	21 (15%)	9 (10.5%)	12 (22.2%)
December	12 (8.6%)	7 (8.1%)	5 (9.3%)	1 (0.7%)	1 (1.2%)	0 (0%)
January	14 (10%)	11 (12.8%)	3 (5.5%)	2 (1.4%)	1 (1.2%)	1 (1.9%)
February	3 (2.1%)	0 (0%)	3 (5.5%)	0 (0%)	0 (0%)	0 (0%)
March	0 (0%)	0 (0%)	0 (0%)	0 (0%)	0 (0%)	0 (0%)
Last season vaccination (Yes)	106 (75.7%)	78 (90.7%)	28 (51.9%)	140 (100%)	86 (100%)	54 (100%)
HAI composite score	4.99 (5.62)	2.58 (2.7)	8.81 (6.84)	2.88 (2.26)	2.97 (2.33)	2.74 (2.17)

*Standard deviations/percentages in parentheses for continuous/categorical variables*.

### Assessment of Changes

**Figure 3:**
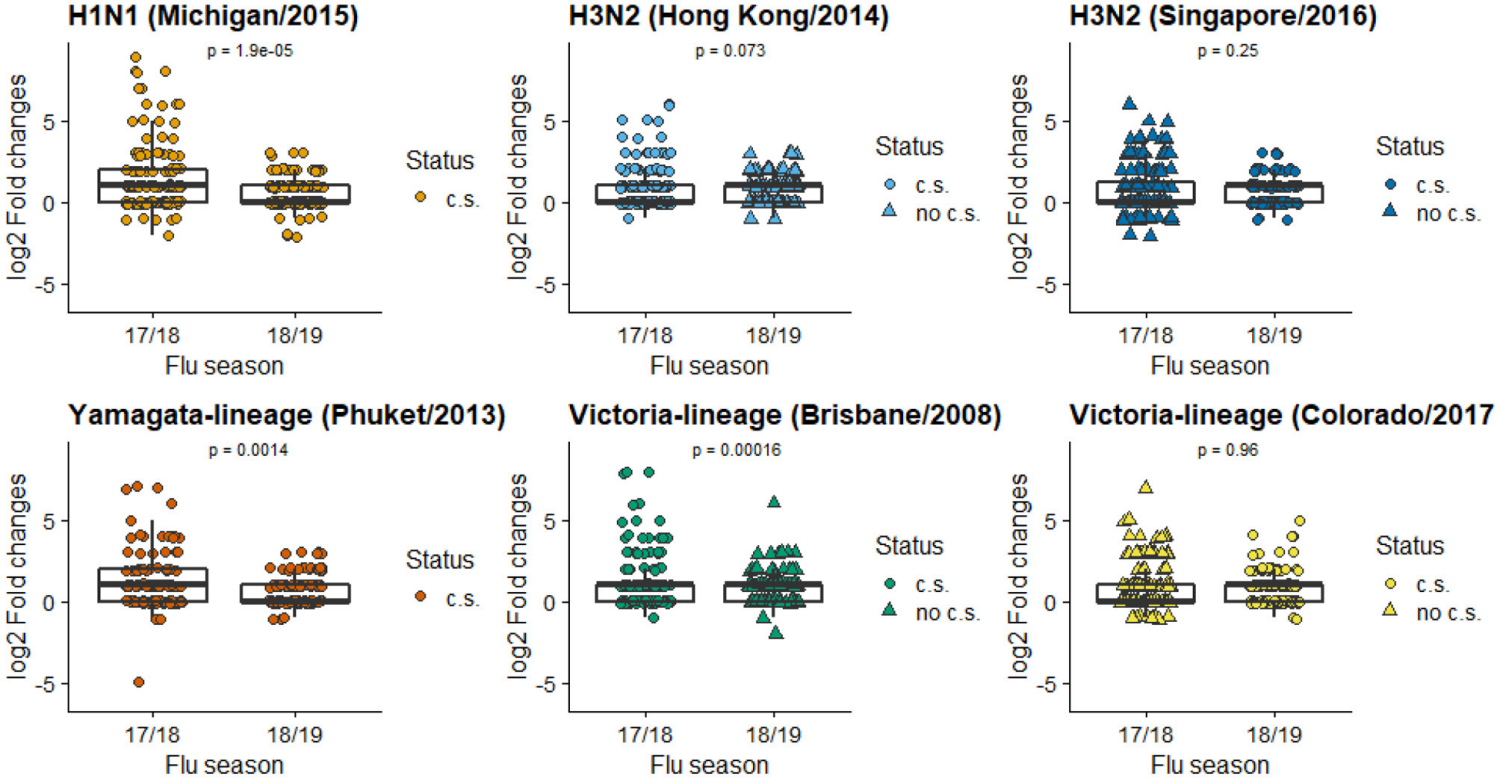
Mean folds changes in HAI titers for each vaccine component strain. (c.s. in current season's vaccine formulation; no c.s. not in current season's vaccine formulation). The Y axis is the log2 transformed fold of changes in HAI titers pre- and post-vaccination, calculated by $log2\left(\frac{HAI_{post}}{HAI_{pre}}\right)$.

**Figure 4:**
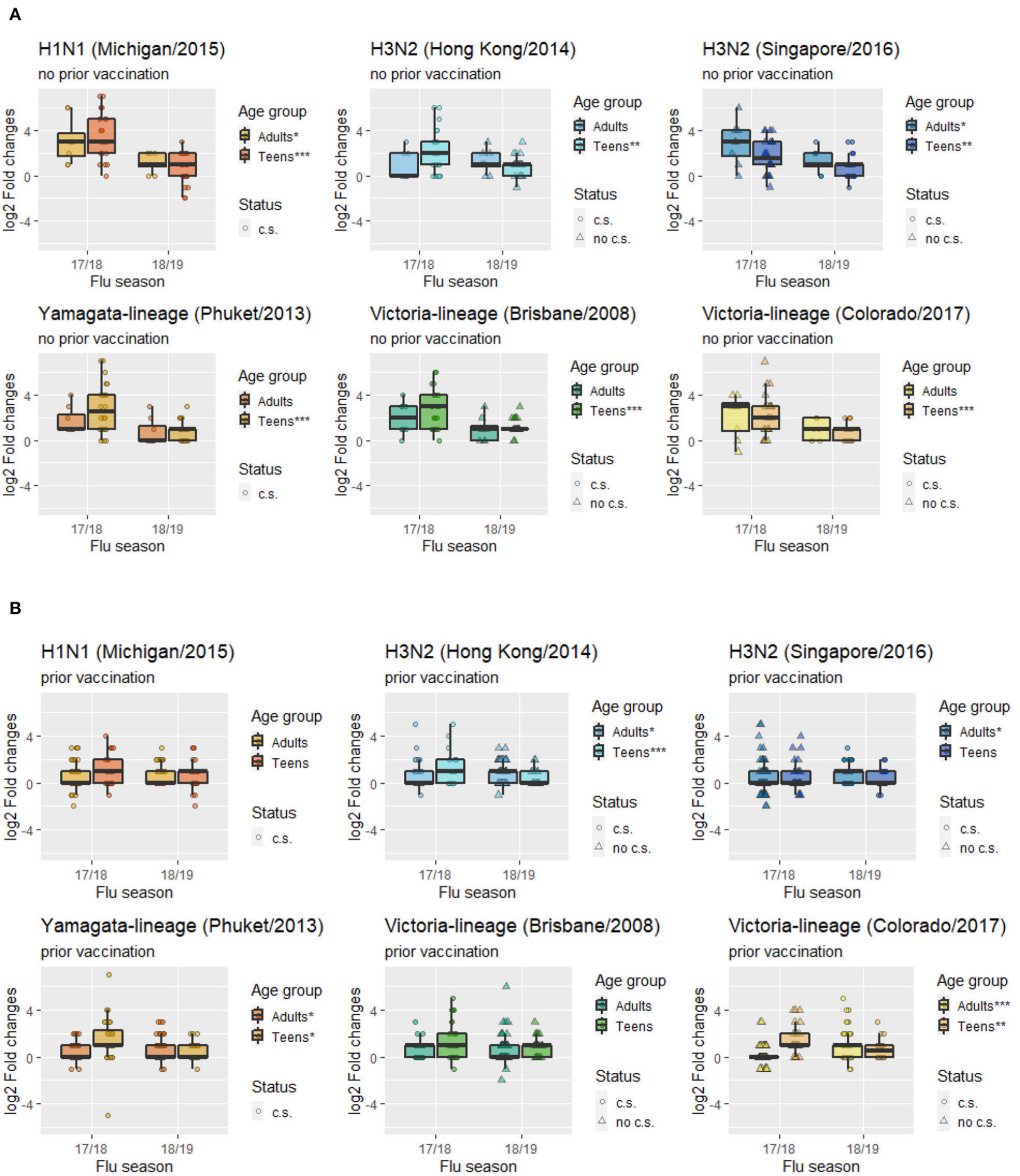
Mean folds changes in HAI titers for each vaccine component strain stratified by age groups and prior vaccination status. **(A)** Subgroup with prior vaccination. **(B)** Subgroup with no prior vaccination. (c.s. in current season's vaccine formulation; no c.s. not in current season's vaccine formulation; ***p < 0.001, **p < 0.01, *p < 0.05).

Box plots of HAI composite scores for the total sample and each age subgroups were also shown in Figure 1. Paired *T*-tests were performed to detect the differences in the mean HAI composite scores from year 1 to year 2. The scores for the total sample (*t* = 4.26, *df* = 139, *p* < 0.001) and the teenagers group (*t* = 6.44, *df* = 53, *p* < 0.001) were significantly decreased in the second year, while the changes in the adults were not statistically significant (*t* = −1.14, *df* = 85, *p* = 0.26). When stratified by prior vaccination (Figure 2), significant declines in the second year were observed in participants without prior vaccination (*t* = 6.78, *df* = 33, *p* < 0.001), but not in those with prior vaccination (*t* = 0.70, *df* = 105, *p* = 0.49). The mean HAI composite scores of the adults increased in those with prior vaccination (*t* = −2.56, *df* = 77, *p* = 0.01) but decreased in those without previous vaccination (*t* = 2.61, *df* = 7, *p* = 0.04). Meanwhile, significant declines were observed in teenagers regardless of their vaccine history (with prior vaccination: *t* = 3.01, *df* = 27, *p* = 0.006; without prior vaccination: *t* = 6.70, *df* = 25, *p* < 0.001). We further compared the yearly mean fold changes of HAI titers (log2 scale) in each influenza virus strain included in the 2 years (Figure 3). The average folds changes of Michigan/2015 (H1N1) (*t* = 4.43, *df* = 139, *p* < 0.001) and Phuket/2013 (Yamagata) (*t* = 3.26, *df* = 139, *p* = 0.001), two strains included in both years' vaccine formula, declined significantly in the second year. For H3N2 where the strains included in the vaccine for each flu season had a change, no statistically significant differences were detected for Hong Kong/2014 (H3N2) (*t* = 1.809, *df* = 139, *p* = 0.07) and Singapore/2016 (H3N2) (*t* = 1.15, *df* = 139, *p* = 0.25) in terms of yearly fold changes of HAI titers between the consecutive years. The influenza B/Victoria strain in the vaccine changed between the 2 years as well. Brisbane/2008 (Victoria) (*t* = 3.88, *df* = 139, *p* < 0.001) had significantly different annual fold changes between 17/18 and 18/19, but no such difference was observed for Colorado/2017 (Victoria) (*t* = −0.05, *df* = 139, *p* = 0.96). We further explored these changes stratified by age group and prior vaccination status (Figures 4A,B). An overall decline trend was observed in teenagers regardless of their previous year vaccination, and such declines were statistically significant across all strains for those vaccinated in 2016–2017. For adults with prior vaccination, the changes were mostly insignificant, with significant declines observed in Michigan/2015 (H1N1) and Singapore/2016 (H3N2). Interestingly, we also noticed an increasing trend of such fold changes in most strains among adults vaccinated in 2016–2017. These results were generally consistent with the overall changes in the HAI composite scores shown in Figure 2.

Stratified analyses in teenagers with and without prior vaccination revealed that among the teenagers (16 subjects) who shad a much higher level of antibody responses to the 2017–2018 vaccination than the rest of group, 12 of them did not receive influenza vaccination in the 2016–2017 flu season (Supplementary Figure 1). This pattern is consistent with the results in Figure 2 showing that teenagers without prior vaccination had higher HAI composite scores in 2017–2018. The same pattern was not observed in the subsequent year where every participant was vaccinated in the previous season. To better understand the potential impact of repeated vaccination in teenagers, we conducted an additional analysis to include teenagers who participated in the 2018–2019 season but were not enrolled in the 2017–2018 season. Supplementary Table 5 provides the 2018–2019 season descriptive statistics for teenagers repeatedly enrolled in the 2 consecutive seasons and those newly enrolled for the first time in the 2018–2019 season. While there were 51% of newly enrolled teenagers reported to be vaccinated in the 2017–2018 flu season, their average HAI composite score was still significantly higher than that from the re-enrolled teenagers. A further stratification of the newly enrolled teenagers by their previous year vaccination status suggests that those who were vaccinated in the previous season had a significantly lower mean HAI composite score (4.10 vs. 11.60).

### Multivariate Analysis

In the linear regression analysis for all 140 participants, baseline HAI titers, age, BMI, prior vaccination in 2016–2017, and flu season 18/19 showed significant negative associations with HAI composite scores (Table 2). The negative coefficient for the flu season 18/19 suggested a decline in vaccine-elicited antibody responses between the years. In addition, the interaction term of age and BMI were positively significant. Similar associations between the host factors and HAI composite scores were found in the logistic regression model when the outcome was dichotomized (Supplementary Table 2). In the stratified analyses with different age groups, baseline HAI titers, history of vaccination, and flu season 18/19 significantly correlated with HAI composite scores in both adults and teenagers (Table 2). BMI and the interaction term of age and BMI were only significant in adults. Results from the logistic regression models were generally in consist with the findings from the linear models (Supplementary Table 2).

**Table 2:** Coefficient estimates of host variables fitted in linear model with GEE.

	**Overall (*n* = 140)**	**Adults (*n* = 86)**	**Teens (*n* = 54)**
Baseline HAI titers	**−** 2.33^***^ **(−** **2.79**, **−** **1.88)**	**−** 1.02^***^ **(−** **1.32**, **−** **0.72)**	**−** 3.20^***^ **(−** **3.84**, **−** **2.57)**
Age	**−** 0.21^**^ **(−** **0.34**, **−** **0.08)**	**−** 0.15* **(−** **0.27**, **−** **0.02)**	0.82 (−2.22, 3.87)
Sex (Male)	−0.08 (−1.50, 1.35)	0.29 (−1.19, 1.77)	−0.44 (−15.11, 14.23)
Race (White)	0.64 (−0.21, 1.49)	0.13 (−0.61, 0.86)	1.14 (−0.27, 2.55)
BMI	**−** 0.25^**^ **(−** **0.41**, **−** **0.09)**	**−** 0.23^*^ **(−** **0.40**, **−** **0.05)**	0.18 (−1.69, 2.06)
Comorbidity (Yes)	−0.32 (−1.14, 0.51)	−0.38 (−1.19, 0.43)	0.18 (−1.03, 1.39)
Prior vaccination (Yes)	**−** 3.34^***^ **(−** **4.22**, **−** **2.47)**	**−** 3.16^***^ **(−** **4.62**, **−** **1.70)**	**−** 3.16^***^ **(−** **4.50**, **−** **1.82)**
Flu season (18/19)	**−** 3.15^***^ **(−** **3.87**, **−** **2.44)**	**−** 0.83^*^ **(−** **1.50**, **−** **0.16)**	**−** 4.36^***^ **(−** **5.57**, **−** **3.16)**
Age*BMI	0.005^*^ **(0.0008, 0.01)**	0.005^*^ **(0.0005, 0.01)**	−0.02 (−0.15, 0.10)
Age* Sex (Male)	−0.003 (−0.05, 0.04)	−0.02 (−0.06, 0.02)	−0.005 (−1.02, 1.01)

*95% confidence interval in parentheses*,

*** *p < 0.001*,

** *p < 0.01*,

* *p < 0.05. The bold values are highlight model parameter estimates that are statistically significant*.

An additional subgroup analysis stratifying younger and older adults with adjusted models using GEE also suggested the potential negative association in younger adults and positive association in older adults (Supplementary Table 4). However, due to the limited sample size in each subgroup, these estimates did not reach statistical significance at the 0.05 level.

## Discussion

Using an HAI composite score as the targeted outcome, we applied GLMs with GEE to assess the associations between vaccine-elicited immune responses and host factors, both repeatedly measured in a human cohort of volunteers who were vaccinated in 2017–2018 and 2018–2019. Overall, HAI composite scores declined significantly from year 1 to year 2 in teenagers but remained steady in adults, likely due to a much higher proportion of adults already receiving vaccination in the previous season. Adults who received vaccination in the previous season had an increase in antibody responses after repeated vaccination, suggesting the benefit of annual vaccination. However, teenagers tend to have lower antibody boosts regardless of their previous vaccination status, implying that a different vaccination strategy might be more beneficial for them. A comparison of mean HAI composite scores in 2018–2019 between the repeatedly enrolled teenagers and those newly enrolled in 2018–2019 also supports the hypothesis that repeated vaccination resulted in reduced immune responses in teenagers (Supplementary Table 5).

In terms of subtype of influenza virus, the mean fold changes in HAI titers between pre- and post-vaccination dropped significantly in the second flu season for those strains included in both years' vaccine formula (H1N1, B/Yamagata), suggesting a repeated vaccination with the same H1N1 and B/Yamagata strains was associated with reduced boosting of immune responses. Mean fold changes to the H3N2 vaccine strains remained at a moderate level without significant differences between the 2 years. The A/Hong Kong/2014 strain included in 2017–2018 was also included in the prior 2016–2017 season but replaced by A/Singapore/2016 in 2018–2019. For B/Victoria, a reduced boost was observed for the B/Brisbane/2008 after it was replaced by B/ Colorado/2017 in the second year, suggesting a lower level of cross-protective immune responses for B/Brisbane/2008. Slight increases were observed in adults with prior vaccination in 4 strains, while declines were observed across all strains among teenagers regardless of their previous year vaccination status. Vaccine-elicited antibody responses to repeated inoculations appear to be quite different between adults and teenagers. We believe repeated vaccination at least partially explains for the decline in HAI composite scores for the H1N1 and B/Phuket strain that were included in both vaccines. Based on the antigenic distance hypothesis, vaccine effectiveness tends to be low when the past and current vaccine strains are similar (24, 25). Some recent studies have also observed reduced vaccine effectiveness in repeat vaccines (25, 26). Meanwhile, there is no change (nor elevation) in the strains that were changed between the two seasons, as those strains were not repeatedly included in the vaccine formula, and thus not subjected to the same level of reduced effectiveness.

There was no statistical difference in HAI composite scores for H3N2/Singapore and B/Victoria/Colorado between 2017–2018 and 2018–2019. However, Figures 4A,B revealed that this might be due to a mixture pattern of those who were vaccinated in the 2016–2017 season vs. those not vaccinated, i.e., adults who were vaccinated in 2016–2017 tend to have deficient cross-reactivities in 2017–2018, but then in 2018–2019 their responses were higher after vaccination (Figure 4A). Meanwhile, both adults and teenagers who were not vaccinated in 2016–2017 had relatively high cross-reactivities for H3N2/Singapore and B/Victoria/Colorado in 2017–2018, but then in 2018–2019 their responses were lower after vaccination (Figure 4B). In addition, a recent study suggested that H1 virus or type B influenza pre-exposure has a negative correlation with the H3-specific post-vaccination response (27). Specifically, prior exposure to H1 virus may significantly affect cross-reactivities of antibody responses elicited by H3 virus as long as the H1 virus is in circulation. This finding may help explain the low HAI responses of H3N2/Singapore in adults with prior vaccination in the 2017–2018 flu season.

In the general cohort, our findings suggest that baseline HAI titers, age, BMI, prior vaccination in 2016–2017, and flu season were significantly associated with the repeatedly measured HAI composite scores in consecutive years. All five variables were negatively associated with the HAI composite scores, indicating lower vaccine-elicited antibody responses in participants who had higher baseline titers, were older, with higher BMIs, or received flu shots in the previous flu season. There was also an overall decline in the 2018–2019 season after other variables were adjusted. The negative impacts of high baseline antibody levels and previous vaccination have been established in the literature (3, 6–8, 28). The negative aging effect on immune response found in our study is also consistent with earlier studies showing that antibody responses to influenza vaccines in the elderly were less likely to reach seroprotection and seroconversion than young adults (8). In addition, a lower rate of seroprotection in the elderly was observed against influenza A and B with repeated vaccinations (29, 30).

Obesity has been considered a risk factor of influenza infection, possibly through impairing human immune response as well as the effectiveness of vaccination (14, 31). The risk of developing influenza and influenza-like illness were twice in vaccinated obese subjects compared with healthy weight subjects (18). Pathological mechanisms related to obesity could have delayed and blunted antiviral responses to influenza virus infection that may increase the risk of severe disease (16, 32). Serological studies showed that H1N1 vaccines could induce higher initial serologic responses among obese adults, but greater declines in their antibody titers were observed in the follow-up (14–16). However, no studies were conducted to establish the association between BMI and the overall vaccine-elicited antibody responses in a repeated vaccination setting, and its interactive effect with age has not been well-studied. In our analysis, the association between BMI and the repeatedly measured overall induced immune responses differed with age. BMI was not identified as a risk factor for teenagers in our study. We did, however, find that BMI plays an important role in adults, and observed different patterns for young and older adults. Generally, younger adults with higher BMIs tend to have fewer folds' increases in total HAI responses than those with low or normal BMIs, but the association reversed for the elderly, with a turning point around the age of 50. In study participants 50 years and above, higher BMIs were associated with increasing folds of change in HAI titers after vaccination. However, a recent study illustrated that the risk of having influenza in relation to BMI is similar when comparing middle-aged and older adults (31).

Subsequent analyses were conducted stratifying the study population by adults and teenagers. Prior vaccination remained a significant predictor for lower HAI scores in both groups, suggesting that those who had flu shots in the previous season, regardless of their ages, are less likely to respond well to annual flu vaccinations in the following seasons. This finding is consistent with previous studies indicating that prior vaccination status significantly influenced the HAI titers levels from post-vaccination (3, 4). The interactive effect between age and BMI was only observed in adults. This is perhaps due to the limited sample size and the lack of diversities in both age and BMI values among the teenagers. The age range for the teenagers was between 12 and 17 years old, and their BMI values were mostly at the normal level, with very few exceptions presenting overweight or obesity. The insufficient heterogeneity of BMI values in the enrolled teenage group prohibited a further investigation on its interaction with age in the logistic regression.

An important strength of the performed analyses is the appropriate adjustment of the correlation among repeated measurements by GEE in a longitudinal analysis. Despite of the many longitudinal studies that assessed the relationships between human immune responses and host factors, very few adjusted for the correlation of repeated antibody measurements from the same subject (17, 18), and neither of them used a comprehensive measure to assess the overall induced antibody levels across multiple flu strains. Instead of focusing on the individual HAI titers considering each single strain of influenza virus, an HAI composite score combining multiple strains was adopted in our study to evaluate the overall vaccine-elicited human immune responses. Such an index allows a more flexible assessment of immune responses to repeated vaccinations when one or more components in the vaccine formula change between the years. In addition, the inclusion of data from a cohort of teenagers in our study provides valuable insights into an understudied population.

Our study also has several limitations. The statistical power of the conducted analyses was limited by the number of participants who re-enrolled in our cohort for consecutive flu seasons, although the repeated measurements contributed valuable additional data points for the assessment of annual vaccinations. Our study sample was dominated by white participants. While we made the comparison between white and non-white participants, a further detailed analysis on the more vulnerable racial groups was not performed due to the limited number of subjects in those groups. Our investigation of the understudied teenager population was somewhat restricted by the lack of heterogeneity in BMI and comorbidity among the enrolled sample. In addition, the current study only includes data from 2 consecutive vaccinations, comparing immunity for more than two seasons may provide further insights into the analysis. However, including more consecutive years would also further reduce the sample size based on the study focus on subjects with repeated vaccinations in consecutive seasons. For all the aforementioned reasons, future cohort studies with larger sample sizes and longer observation durations are highly demanded. Meanwhile, HAI is just one correlate of protection for influenzas, and others (e.g., neutralizing antibodies or T-cells) also help protect. However, we did not have those data in the current cohort study.

In conclusion, our study found distinct vaccine-elicited immune responses between adults and teenagers when both were repeatedly vaccinated in consecutive years. It also provided further supports for the associations between several host factors and antibody responses in a repeated vaccination setting. Notably, we found an interactive effect of age and BMI in the overall population and the adults. Future studies with a larger enrollment of teenaged participants representing a broader range of BMIs may provide additional insights into the interactive effect in the younger age group.

## Data Availability Statement

The raw data supporting the conclusions of this article will be made available by the authors, without undue reservation.

## Ethics Statement

The studies involving human participants were reviewed and approved by the Institutional Review Board of the University of Georgia. Written informed consent to participate in this study was provided by the participants' legal guardian/next of kin.

## Author Contributions

M-HS: literature search, figures, data analysis, data interpretation, and writing. YS: study design, data analysis, data interpretation, figures, and writing. AH and TR: study design, data interpretation, and writing. JB: data interpretation and writing. All authors contributed to the article and approved the submitted version.

## Conflict of Interest

The authors declare that the research was conducted in the absence of any commercial or financial relationships that could be construed as a potential conflict of interest.
