# Supplementary material for: Longitudinal Assessment of Immune Responses to Repeated Annual Influenza Vaccination in a Human Cohort of Adults and Teenagers
Source: Front Immunol. 2021 Mar 3;12:642791. doi: 10.3389/fimmu.2021.642791 (PMC7965973; doi:10.3389/fimmu.2021.642791)

**Longitudinal Assessment of Immune Responses to Repeated Annual Influenza Vaccination in a Human Cohort of Adults and Teenagers**

**Supplementary Appendix**

**Contents**

Table S1. Descriptive statistics of UGA cohort study by years

Table S2. Descriptive statistics of adults re-enrolled in both seasons

Table S3. Odds ratio estimates of host variables fitted in logistic model with GEE

Table S4. Coefficient estimates of host variables fitted in linear model with GEE stratified by younger and older adults

Table S5. Descriptive statistics of teenagers re-enrolled in both seasons and newly enrolled in 2018-2019

Figure S1. HAI Composite Scores from Teenagers with and without Prior Vaccination

**Table S1. Descriptive statistics of UGA cohort study by years**

|  | **2017-2018 (single year)** | **2018-2019 (single year)** |
| --- | --- | --- |
| **Sample sizes** | 255 | 242 |
| **Age** | 31.96 (18.09) | 21.14 (16.12) |
| *Adults (>=18 years)* | 183 (71.8%) | 92 (38%) |
| *Teenagers (<18 years)* | 72 (28.2%) | 150 (62%) |
| **BMI** | 26.62 (6.4) | 24.45 (6.04) |
| **Sex (Male)** | 113 (44.3%) | 105 (43.4%) |
| **Race** |  |  |
| *White* | 205 (80.4%) | 203 (83.9%) |
| *African American* | 19 (7.5%) | 11 (4.5%) |
| *Other* | 31 (12.1%) | 28 (11.6%) |
| **Comorbidity (Yes)** | 59 (23.1%) | 53 (21.9%) |
| **Month of Vaccination** |  |  |
| *September* | 25 (9.8%) | 93 (38.4%) |
| *October* | 68 (26.7%) | 93 (38.4%) |
| *November* | 87 (34.1%) | 40 (16.5%) |
| *December* | 24 (9.4%) | 11 (4.5%) |
| *January* | 44 (17.3%) | 4 (1.7%) |
| *February* | 7 (2.7%) | 1 (0.4%) |
| *March* | 0 (0%) | 0 (0%) |
| **Last season vaccination (Yes)** | 181 (71%) | 193 (79.8%) |
| **HAI composite score** | 5.18 (5.46) | 4.83 (4.7) |

(standard deviations/percentages in parentheses for continuous/categorical variables)

**Table S2. Descriptive statistics of adults re-enrolled in both seasons**

|  | **2017-2018** | | **2018-2019** | |
| --- | --- | --- | --- | --- |
|  | **Age < 50** | **Age >= 50** | **Age < 50** | **Age >= 50** |
| **Sample sizes** | 60 | 26 | 60 | 26 |
| **Age** | 29 (8.58) | 61.19 (6.60) | 30.13 (8.66) | 61.69 (6.67) |
| **BMI** | 26.63 (5.17) | 30.86 (4.85) | 26.59 (5.28) | 30.79 (4.75) |
| **Sex (Male)** | 22 (36.7%) | 12 (46.2%) | 22 (36.7%) | 12 (46.2%) |
| **Race** |  |  |  |  |
| *White* | 48 (80%) | 21 (80.8%) | 48 (80%) | 21 (80.8%) |
| *African American* | 2 (3.3%) | 3 (11.5%) | 2 (3.3%) | 3 (11.5%) |
| *Other* | 10 (16.7%) | 2 (7.7%) | 10 (16.7%) | 2 (7.7%) |
| **Comorbidity (Yes)** | 11 (18.3%) | 13 (50%) | 10 (16.7%) | 12 (46.2%) |
| **Month of Vaccination** |  |  |  |  |
| *September* | 6 (10%) | 5 (19.2%) | 28 (46.7%) | 16 (61.5%) |
| *October* | 18 (30%) | 9 (34.6%) | 24 (40%) | 7 (26.9%) |
| *November* | 22 (36.7%) | 8 (30.8%) | 6 (10%) | 3 (11.5%) |
| *December* | 4 (6.7%) | 3 (11.5%) | 1 (1.7%) | 0 (0%) |
| *January* | 10 (16.7%) | 1 (3.8%) | 1 (1.7%) | 0 (0%) |
| *February* | 0 (0%) | 0 (0%) | 0 (0%) | 0 (0%) |
| **Last season vaccination (Yes)** | 55 (91.7%) | 23 (88.5%) | 60 (100%) | 26 (100%) |
| **HAI composite score** | 2.42 (2.59) | 2.96 (2.96) | 2.8 (2.23) | 3.35 (2.54) |

(standard deviations/percentages in parentheses for continuous/categorical variables)

**Table S3. Odds ratio estimates of host variables fitted in logistic model with GEE**

|  | Overall (n=140) | Adults (n=86) |
| --- | --- | --- |
| Baseline HAI titers | ${\begin{aligned} \boldsymbol{0.29} \\ \boldsymbol{(0.20, 0.42)} \end{aligned}}^{\boldsymbol{***}}$ | $\boldsymbol{0.33}^{\boldsymbol{***}}$  $\boldsymbol{(0.20, 0.54)}$ |
| Age | ${\begin{aligned} \boldsymbol{0.76} \\ \boldsymbol{(0.66, 0.86)} \end{aligned}}^{\boldsymbol{***}}$ | $\boldsymbol{0.69}^{\boldsymbol{***}}$  $\boldsymbol{(0.55, 0.86)}$ |
| Sex (Male) | 0.30  (0.07, 1.27) | 0.91  (0.05, 15.92) |
| Race (White) | 0.76  (0.37, 1.56) | 0.64  (0.25, 1.61) |
| BMI | ${\begin{aligned} \boldsymbol{0.67} \\ \boldsymbol{(0.56, 0.80)} \end{aligned}}^{\boldsymbol{***}}$ | $\boldsymbol{0.55}^{\boldsymbol{**}}$  $\boldsymbol{(0.38, 0.79)}$ |
| Comorbidity (Yes) | 0.58  (0.20, 1.68) | 0.85  (0.20, 3.66) |
| Prior vaccination (Yes) | ${\begin{aligned} \boldsymbol{0.14 (} \\ \boldsymbol{0.06, 0.32)} \end{aligned}}^{\boldsymbol{***}}$ | $\boldsymbol{0.05}^{\boldsymbol{***}}$  $\boldsymbol{(0.01, 0.30)}$ |
| Flu season (18/19) | ${\begin{aligned} \boldsymbol{0.48} \\ \boldsymbol{(0.26, 0.89)} \end{aligned}}^{\boldsymbol{*}}$ | 0.73  (0.31, 1.70) |
| Age*BMI | ${\begin{aligned} \boldsymbol{1.01} \\ \boldsymbol{(1.005, 1.01)} \end{aligned}}^{\boldsymbol{***}}$ | $\boldsymbol{1.01}^{\boldsymbol{**}}$  $\boldsymbol{(1.005, 1.02)}$ |
| Age* Sex (Male) | 1.02  (0.97, 1.06) | 0.99  (0.92, 1.07) |

(95% confidence interval in parentheses, ‘***’: p<0.001, ‘**’: p< 0.01, ‘*’: p< 0.05)

**Table S4. Coefficient estimates of host variables fitted in linear model with GEE stratified by younger and older adults**

|  | **Age < 50**  (n=60) | **Age >= 50** (n=26) |
| --- | --- | --- |
| Baseline HAI titers | $\boldsymbol{-1.13}^{\boldsymbol{***}}$**(-1.49, -0.78)** | -0.52 (-1.08, 0.05) |
| Sex (Male) | -0.19 (-0.95, 0.58) | -0.89 (-2.16, 0.37) |
| Race (White) | 0.45 (-0.50, 1.40) | -0.17 (-1.05, 0.71) |
| BMI | -0.06 (-0.15, 0.02) | 0.02 (-0.12, 0.16) |
| Comorbidity (Yes) | -0.19 (-1.30, 0.91) | -0.75 (-1.86, 0.36) |
| Prior vaccination (Yes) | $\boldsymbol{-2.69}^{\boldsymbol{**}}$**(-4.55, -0.83)** | $\boldsymbol{-3.73}^{\boldsymbol{**}}$**(-5.18, -2.28)** |
| Flu season (18/19) | $\boldsymbol{-0.84}^{\boldsymbol{*}}$**(-1.56, -0.12)** | -0.38 (-2.04, 1.28) |

(95% confidence interval in parentheses, ‘***’: p<0.001, ‘**’: p< 0.01, ‘*’: p< 0.05)

**Table S5. Descriptive statistics of teenagers re-enrolled in both seasons and newly enrolled in 2018-2019**

|  | **2018-2019** | | | | | |
| --- | --- | --- | --- | --- | --- | --- |
|  | **Teens (Repeatedly Enrolled)** |  | | **Teens (Newly Enrolled in 2018-2019)** | | |
|  |  | |  | All | Prior vaccination | No Prior vaccination |
| **Sample sizes** | 54 | |  | 96 | 49 | 47 |
| **Age** | 15.13 (1.29) | |  | 14.26 (1.71) | 14.22 (1.74) | 14.30 (1.69) |
| **BMI** | 22.16 (3.61) | |  | 22.54 (6.21) | 22.35 (6.56) | 22.74 (5.90) |
| **Sex (Male)** | 23 (42.6%) | |  | 46 (47.9%) | 25 (51.0%) | 21 (44.7%) |
| **Race** |  | |  |  |  |  |
| *White* | 45 (83.3%) | |  | 85 (88.5%) | 44 (89.8%) | 41 (87.2%) |
| *African American* | 1 (1.9%) | |  | 5 (5.2%) | 1 (2.0%) | 4 (8.5%) |
| *Other* | 8 (14.8%) | |  | 6 (6.3%) | 4 (8.2%) | 2 (4.3%) |
| **Comorbidity (Yes)** | 14 (25.9%) | |  | 17 (17.7%) | 8 (16.3%) | 9 (19.1%) |
| **Month of Vaccination** |  | |  |  |  |  |
| *September* | 17 (31.5%) | |  | 29 (30.2%) | 13 (26.5%) | 16 (34.0%) |
| *October* | 24 (44.4%) | |  | 35 (36.5%) | 19 (38.8%) | 16 (34.0%) |
| *November* | 12 (22.2%) | |  | 19 (19.8%) | 14 (28.6%) | 5 (10.6%) |
| *December* | 0 (0%) | |  | 10 (10.4%) | 3 (6.1%) | 7 (14.9%) |
| *January* | 1 (1.9%) | |  | 2 (2.1%) | 0 (0%) | 2 (4.3%) |
| *February* | 0 (0%) | |  | 1 (1.0%) | 0 (0%) | 1 (2.1%) |
| *March* | 0 (0%) | |  | 0 (0%) | 0 (0%) | 0 (0%) |
| **Last season vaccination (Yes)** | 54 (100%) | |  | 49 (51.0%) | 49 (100%) | 0(0%) |
| **HAI composite score** | 2.74 (2.17) | |  | 7.77 (5.83) | 4.10 (4.07) | 11.60 (4.86) |

(standard deviations/percentages in parentheses for continuous/categorical variables)

**Figure S1. HAI Composite Scores from Teenagers with and without Prior Vaccination**


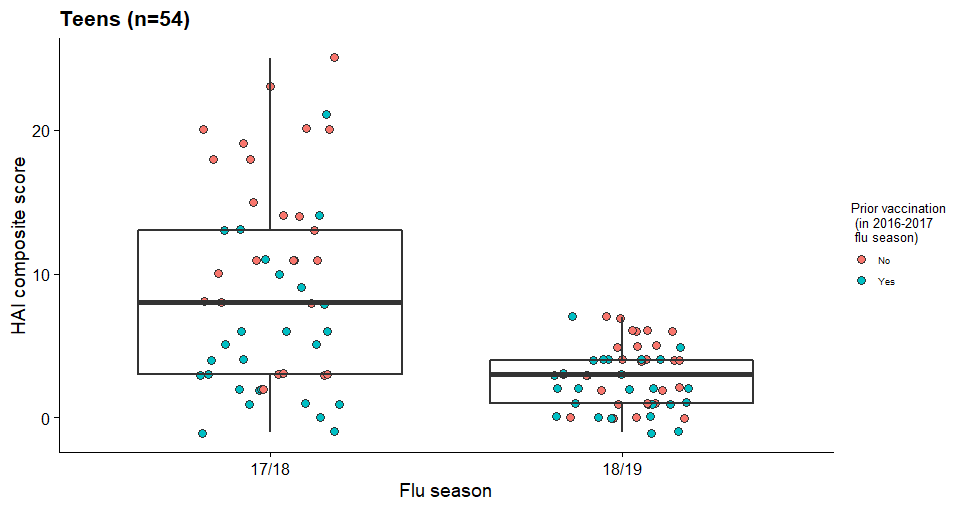

Supplement: Supplementary file 1 [file Data_Sheet_1.docx]
